# Achieving room air quality of room class Ib in the aseptic area using a mobile sterile ventilation unit in a room class II surgical unit

**DOI:** 10.3205/dgkh000521

**Published:** 2024-12-16

**Authors:** Dorothee Boppre, Martin Exner, Colin M. Krüger, Hannes Schuler, Michael Wendt, Julian-Camill Harnoss, Axel Kramer

**Affiliations:** 1 Institute of Hygiene and Environmental Medicine, University Medicine Greifswald, Germany; 2 Institute of Hygiene and Public Health, Bonn, Germany; 3 Department of Surgery, Centre of Robotics, University Hospital Ruedersdorf, Medical University Brandenburg Theodor-Fontane, Ruedersdorf, Germany; 4 Clinic and outpatient clinic for Anaesthesiology and Intensive Care Medicine, University Medicine Greifswald, Germany; 5 Department of General, Visceral and Transplantation Surgery, University Hospital Heidelberg, Germany

**Keywords:** mobile sterile ventilation unit, elimination of microorganisms, elimination of particles, room class Ib, alternative for room ventilation systems

## Abstract

**Introduction:**

Room air class (RC) Ib may be necessary for surgical procedures in aseptic working areas. The aim of the study was to examine whether a mobile, three-stage sterile ventilation unit (MSVU) can replace a room ventilation system (RVS) with turbulent mixed flow (TMF) in the area of the operating field and on the instrument table from hygienic-microbiological point of view.

**Method:**

During 26 surgeries (varicose vein stripping or treatment of umbilical and inguinal hernias), the microbial load was recorded at 4 measuring points (M1–M4) during regular operations by setting up sedimentation plates and measuring the particle concentration. Measuring points M1 and M2 were located at the beginning and the end of the instrument table, measuring point M3 next to the operating field and measuring point M4 outside the sterilely ventilated area approx. 135 cm from the operating field. The measured values were compared with results with simulated, incorrect positioning and with MSVU not switched on.

**Results:**

The number of people and the duration of the operation did not differ between the 3 measurement situations.

The MSVU achieved a significant reduction in the number of sedimented colony-forming units (CFU) at M1 by 88.4%, at M2 by 91.5% and at M3 by 65.2%. At measuring point M4, the values did not differ between MSVU switched on or off. Even with an unacceptably increased distance between the MSVU and the instrument table, the difference at measuring points M1, M2 and M3 was still significant in comparison with MSVU switched off. Coagulase-negative staphylococci were predominantly detected, followed by *Micrococcus luteus* and apathogenic spore-forming bacteria, but Gram-negative bacteria were not detected in any cases. The number of CFU detected fulfils the criteria for conventionally turbulent non-directionally ventilated surgical units with TMF of RC Ib.

The particle count was reduced by an average of 66%. As comparable particle counts were found in the aseptic working area in a separately conducted study in an RC Ib surgical unit, it can be assumed that the results obtained with the MSVU are hygienically safe.

**Conclusion:**

With the MSVU, a reduction of the microbial load and the particle count in the room air was achieved in the area of the operating field and on the instrument table during operation in an RC II surgical unit, which can be categorised as sufficient for operations in RC Ib. With the aid of an MSVU, operations with a high risk of surgical sire infections can also be carried out in surgical units of RC II from hygienic-microbiological point of view.

The MSVU is an organisationally flexible and economically interesting, safe and sustainable option in terms of the microbiological load and particle count in the operating field and instrument table instead of an RVS that ventilates the entire room. In times of increasing outpatientisation of surgical services, MSVU is a promising option for outpatient surgical units in particular.

## Zusammenfassung

**Einleitung:**

Für chirurgische Eingriffe kann im aseptischen Arbeitsbereich die Raumluftklasse (RK) Ib erforderlich sein. Ziel der Studie war es zu überprüfen, ob eine mobile, dreistufige Sterilbelüftungseinheit (mStBE) aus hygienisch-mikrobiologischer Sicht eine Raumlufttechnische Anlage (RLTA) mit turbulenter Mischströmung (TVS) im Bereich des Operationsfelds und auf dem Instrumententisch ersetzen kann.

**Methode:**

Bei 26 Operationen (Varizen-Stripping und Versorgung von Nabel- und Leistenhernien) wurde während des regulären Operationsbetriebs die mikrobielle Belastung an 4 Messpunkten (M1–M4) durch Aufstellen von Sedimentationsplatten und Messung der Partikelkonzentration erfasst. Die Messpunkte M1 und M2 befanden sich am Beginn und am Ende des Instrumententischs, Messpunkt M3 neben dem Operationsfeld und Messpunkt M4 außerhalb des steril belüfteten Bereichs ca. 135 cm vom Operationsfeld entfernt. Die Messwerte wurden mit Ergebnissen bei simulierter, inkorrekter Positionierung und bei nicht eingeschalteter mStBE verglichen.

**Ergebnisse:**

Die Personenzahl und die Operationsdauer unterschieden sich nicht zwischen den 3 Messsituationen.

Durch die mStBE wurde eine signifikante Reduktion der Anzahl sedimentierter Koloniebildender Einheiten (KbE) an M1 um 88,4%, an M2 um 91,5% und an M3 um 65,2% erreicht. Am Messpunkt M4 unterschieden sich die Werte nicht zwischen ein- bzw. ausgeschalteter mStBE. Selbst bei unzulässig vergrößertem Abstand der mStBE zum Instrumententisch war der Unterschied an den Messpunkten M1, M2 und M3 noch signifikant im Vergleich zur abgeschalteten mStBE. Es wurden überwiegend Koagulase-negative Staphylokokken, gefolgt von *Micrococcus* *luteus* und apathogenen sporenbildenden Bakterien, jedoch in keinem Fall Gram-negative Bakterien nachgewiesen. Die Anzahl nachgewiesener KbE erfüllt die Kriterien für konventionell turbulent ungerichtet belüftete Operationseinheiten mit TVS der RK Ib.

Die Partikelzahl wurde durchschnittlich um 66% reduziert. Da in einer separat durchgeführten Untersuchung in einer Operationseinheit der RK Ib vergleichbare Partikelzahlen im aseptischen Arbeitsbereich gefunden wurden, kann von hygienischer Unbedenklichkeit der mit der mStBE erzielten Ergebnisse ausgegangen werden.

**Schlussfolgerung:**

Mit der mStBE wurde im Bereich des Operationsfelds und auf dem Instrumententisch bei Betrieb in einer Operationseinheit der RK II eine Reduktion der mikrobiellen Belastung und der Partikelzahl in der Raumluft erreicht, die für Operationen in der Raumklasse Ib als ausreichend einzustufen ist. Mithilfe einer mStBE können aus hygienisch-mikrobiologischer Sicht auch Operationen mit hohem Risiko für postoperative Wundinfektionen in Operationseinheiten der RK II durchgeführt werden. Die mStBE ist eine organisatorisch flexible und betriebswirtschaftliche interessante, bezogen auf die mikrobiologische Belastung und Partikelzahl im OP-Feld und Instrumententisch unbedenkliche und nachhaltige Option anstelle einer den gesamten Raum belüftenden RLTA. In Zeiten der zunehmenden Ambulantisierung chirurgischer Leistungen ist die mStBE insbesondere für ambulante Operationseinheiten eine aussichtsreiche Option.

## Objective

Operating units are ventilated either with room ventilation systems (RVS) with low-turbulence displacement flow (so-called laminar air flow, LAF), with turbulent mixed flow (TMF) or by means of windows instead of an RVS. The rooms ventilated using RVS are classified as room class (RC) Ia when ventilated with LAF, RC Ib with TMF and RC II with window ventilation. For surgical units with a high or medium risk of developing a surgical site infection (SSI), RC Ib is the standard; for surgical units or procedure rooms for performing operations with a low risk of developing an SSI, RC II is possible as an alternative [1]. 

In RVS with TMF, sterile filtered supply air is introduced into the room in a non-directional, turbulent manner via outlets in the ceiling. In RVS with LAF, directed low-turbulence sterile filtered supply air from the entire ceiling diffuser is directed evenly onto the operating field, the instrument table and the aseptically working surgical team. In both cases, the air flow is directed vertically from top to bottom. With both types of ventilation, a permanent supply air flow creates a relative overpressure to adjacent rooms, preventing air from neighbouring rooms from escaping. The supply of bacteria-free filtered air in the form of LAF or TMF significantly reduces the load of microorganisms and particles in the aseptic work area, whereby the reduction rate for airborne microrganisms is higher with LAF than with TMF [2], [3], [4], [5], [6], [7]. 

The evidence for the influence of both forms of RVS ventilation on the rate of SSI is limited. In older prospective controlled studies, RVS with LAF was shown to have an additional prophylactic effect against SSIs, but this was masked by the effect of simultaneously established PAP [8], [9], [10]. In a prospective cohort study with identical perioperative antibiotic prophylaxis and the use of impermeable surgical gowns and surgical field drapes with high performance quality, the reoperation rate after implantation of hip prostheses was significantly lower when ventilated with LAF [11], as well as in a retrospective study after arterial bypass [12]. However, more recent retrospective controlled studies and reviews have shown no protective effect of LAF compared to TMF [13], [14], [15], [16], [17], [18], [19] and even an increase in SSIs [20]. Even if the studies have limitations, no specific infection-preventing effect can be derived from the use of RVS with LAF based on the study situation [1], [20]. One reason for this may be the drop in the patient’s body temperature below the critical value of 36°C [21] caused by the directed airflow during longer operations, unless the patient is actively warmed, as hypothermia favours the development of SSI [22]. In addition, the unfavourable vertical flow direction from top to bottom during LAF means that turbulence is unavoidable, both from the surgical team and from the operating lamps. As the surgical team is not completely encased in sterile drapes, microorganisms of the skin flora released by turbulence can enter the surgical field. Turbulence is particularly critical if the size of the LAF system is too small (demonstrated in a comparison of sizes 380 cm×120 cm instead of 518 cm×380 cm), because pathogens are then swirled from the floor into the operating area as soon as a team member unintentionally steps out of the flow field, especially partially [5]. However, depending on the size of the surgical unit and the different levels of physical exertion during the procedure, an RVS may be required for fresh air supply and air conditioning, while a separate suction system is also be necessary for TMF if harmful substances (surgical smoke and anaesthetic gases if still in use) are present.

The recommendation of the Commission for Hospital Hygiene and Infection Prevention on the prevention of SSIs states that for operations with low risk of developing an SSI, sufficient window ventilation can be considered as an alternative to RC Ib, provided that the entry of contaminants from outside, e.g. through fly windows, is prevented [1]. Examples include minor eye, ear, nose, throat and maxillofacial surgery, interventional radiological and cardiological surgery and minor skin/subcutaneous surgery. For surgeries with medium and high SSI risk, including visceral surgery, orthopaedic and vascular surgery without permanent implants, major eye, ear, nose, throat and maxillofacial surgery as well as interventional surgeries as extravascularly inserted implants, e.g. pacemakers and surgeries that are likely to have serious consequences, e.g. heart valve replacement, aortic prosthesis, permanent orthopaedic implants and organ transplantation, ventilation with triple-filtered air, i.e. RC Ib, is recommended [1]. However, there is a lack of evidence for RC Ia for surgeries with medium and high SSI risk [1]. Even for RC Ib there is no epidemiological justification; only studies on its efficiency in terms of reducing airborne microorganisms and particles.

As the installation of an RVS is linked to extensive structural requirements with high investment and maintenance costs, requires a high energy input because the entire room is ventilated, and has the disadvantage of vertical flow from top to bottom, a mobile sterile ventilation unit (MSVU) is desirable, which only flows horizontally over the aseptic working areas, i.e. the operating field and the instrument table, with three-stage sterile filtered air. The use of an MSVU also contributes to sustainability, as only the area above the operating field is ventilated instead of the entire room. As MSVUs significantly reduce the microbial and particle load in the aseptic area with overflow [23], [24], [25], [26], it should be hygienically and microbiologically tested whether a three-stage MSVU (prefilter F7, HEPA filter H14 and laminar filter at the outlet of the HEPA filter), which overflows the instrument table and the operating field with LAF, can be used in a RC II operating theatre in the aseptic working area, i.e. above the opened operating field and the instrument table, RC Ib can be achieved.

## Method

### Setting

Between February 2019 and February 2020, an outpatient surgical unit in RC II was investigated to determine whether RC Ib can be achieved by using an MSVU in the aseptic working area. For this purpose, the microbial load and the particle concentration were determined for n=26 surgeries and the temperature and relative humidity were measured during regular surgery. For comparison, the load of microorganisms and particles in the room air was analysed after increasing the distance between the flow outlet of the MSVU and the surgical field during five operations and during two operations when the system was switched off. The measurements were carried out during varicose vein stripping and during the treatment of umbilical and inguinal hernias, as the the normal course of surgery is least disturbed by the measurements during these operations. 

The study was approved by the Ethics Committee of Greifswald University Medicine (ethics vote BB 082/18) and the consent of the surgical practice. The study was conducted in accordance with the Declaration of Helsinki. 

**Figure 1:**
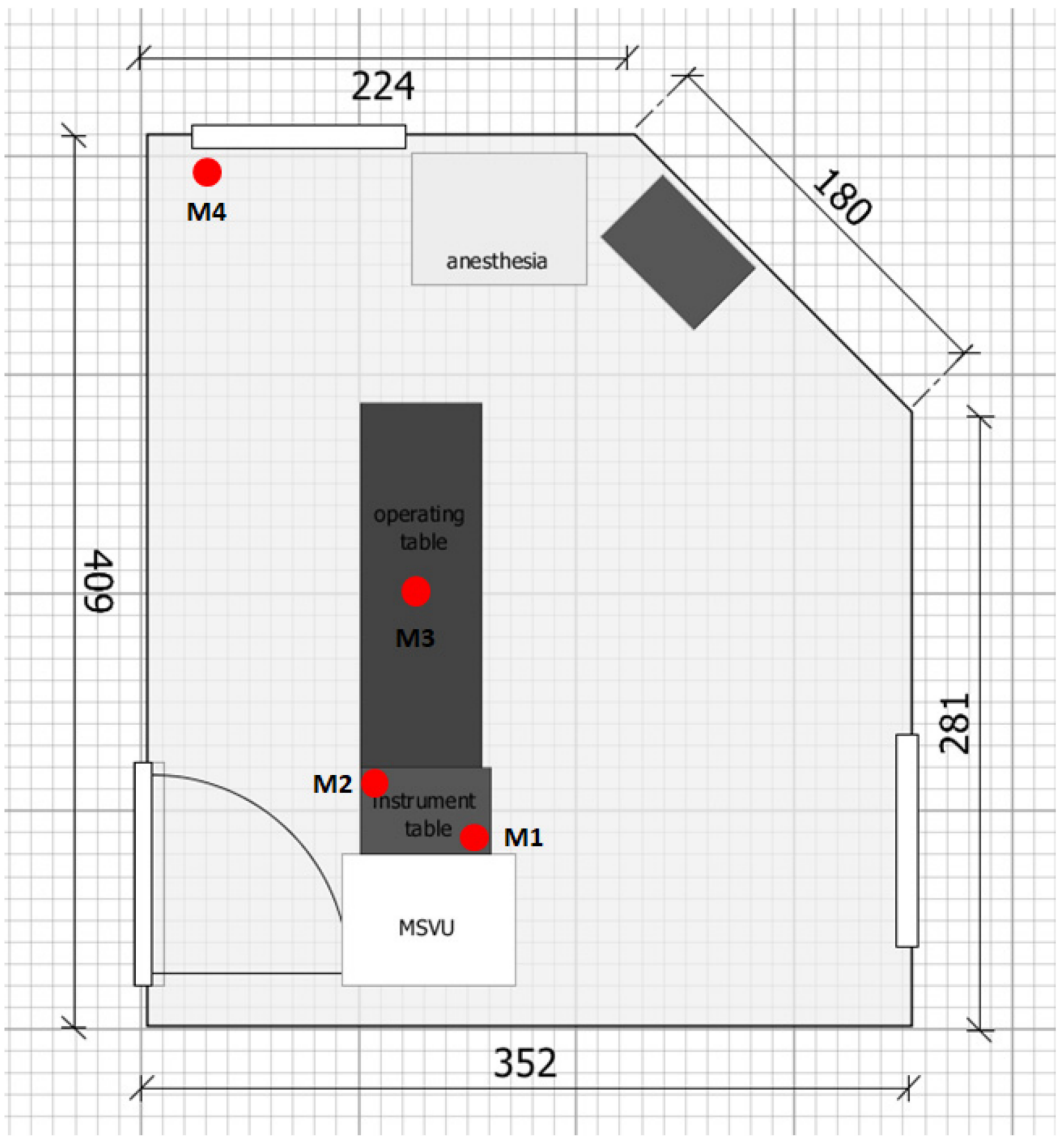
Sketch of the operating unit with arrangement of the measuring points M1 to M4

The operating unit has a volume of 38.02 m^3^ and has no RVS. The room has a door that opens inwards and two gauze-protected windows on the wall opposite the door and on the wall to the left of the door (Figure 1 (Fig. 1)). The preparation room is located in front of the operating unit, where the sterile supplies are stored in a closed area and the patient is prepared for the operation. In addition to the patient, the surgeon, the operating nurse, the anesthesiologist, and the doctoral student were present during the operation to carry out the measurements.

A distinction was made between 3 measurement situations:

- A=Measurement with optimum alignment of the airflow outlet from the MSVU without distance to the instrument table (n=26 surgeries)
- B=Measurement with simulated incorrect positioning of the MSVU with a distance of 40 cm between the airflow outlet and the instrument table (n=5 surgeries)
- C=Measurement with MSVU not switched on as the first measurement before the start of the surgical programme (n=2).

### Devices and materials used

The following were used

- the Operio MSVU (60x45 cm display screen), air flow velocity 0.4–0.5 m/s, air circulation 400 m^3^/h; Toul Meditech AB, Västerås, Sweden),
- the Abakusis particle counter (Markus Klotz GmbH, Bad Liebenzell, Germany) and
- the Testo 400 universal climate meter (Testo SE & Co. KGaA, Titisee-Neustadt, Germany).

Sterile triple-blistered CASO-Agar-ICR 30 ml (casein soya peptone agar; Isolators and Clean Rooms, Ø 90 mm; Merck KGaA, Darmstadt, Germany) were used as sedimentation plates in the aseptic area. Columbia blood agar sedimentation plates (Ø 90 mm, Merck KGaA, Darmstadt, Germany) were used for the measurements in the non-aseptic area. 

**Figure 2:**
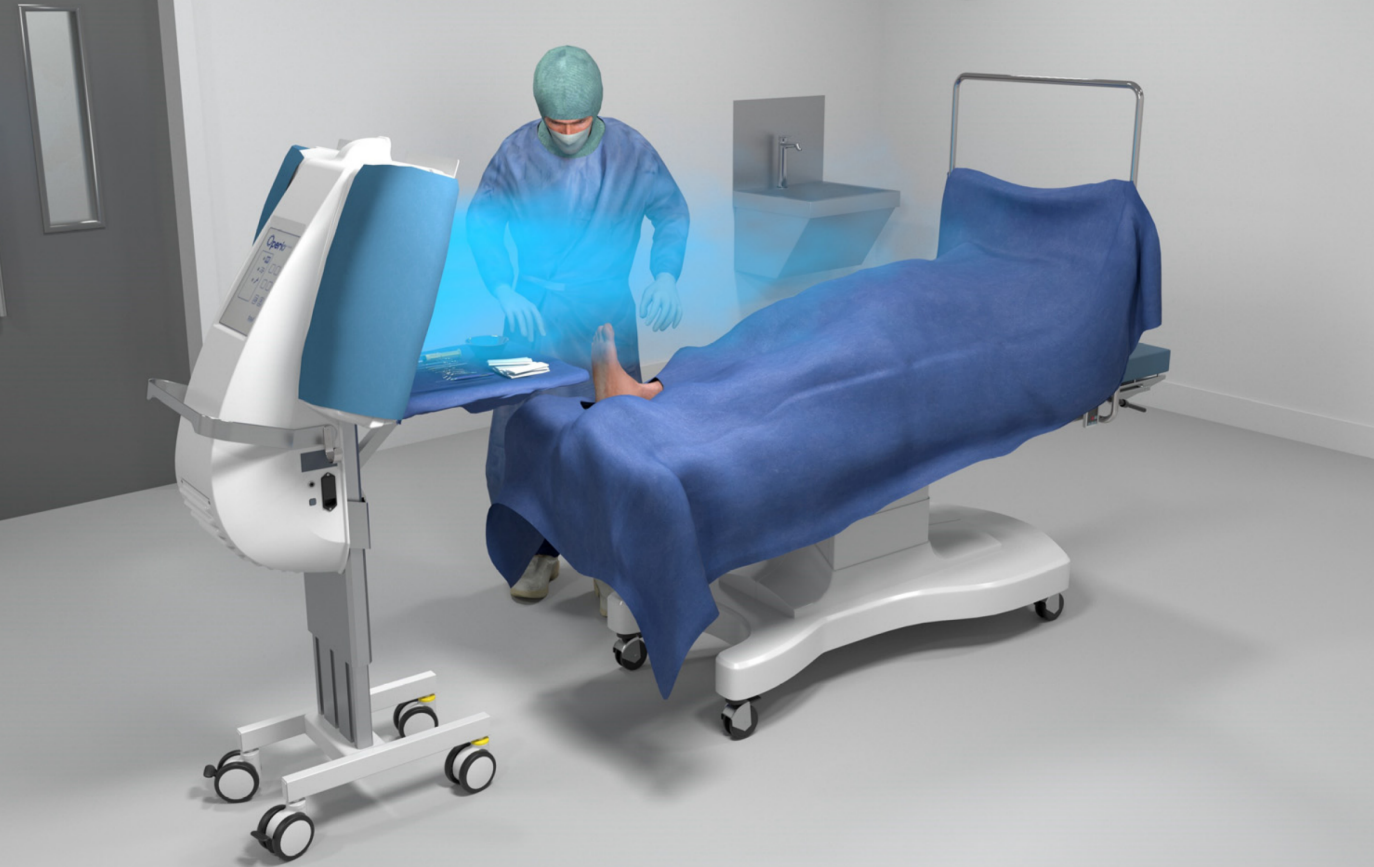
Operation on the foot

**Figure 3:**
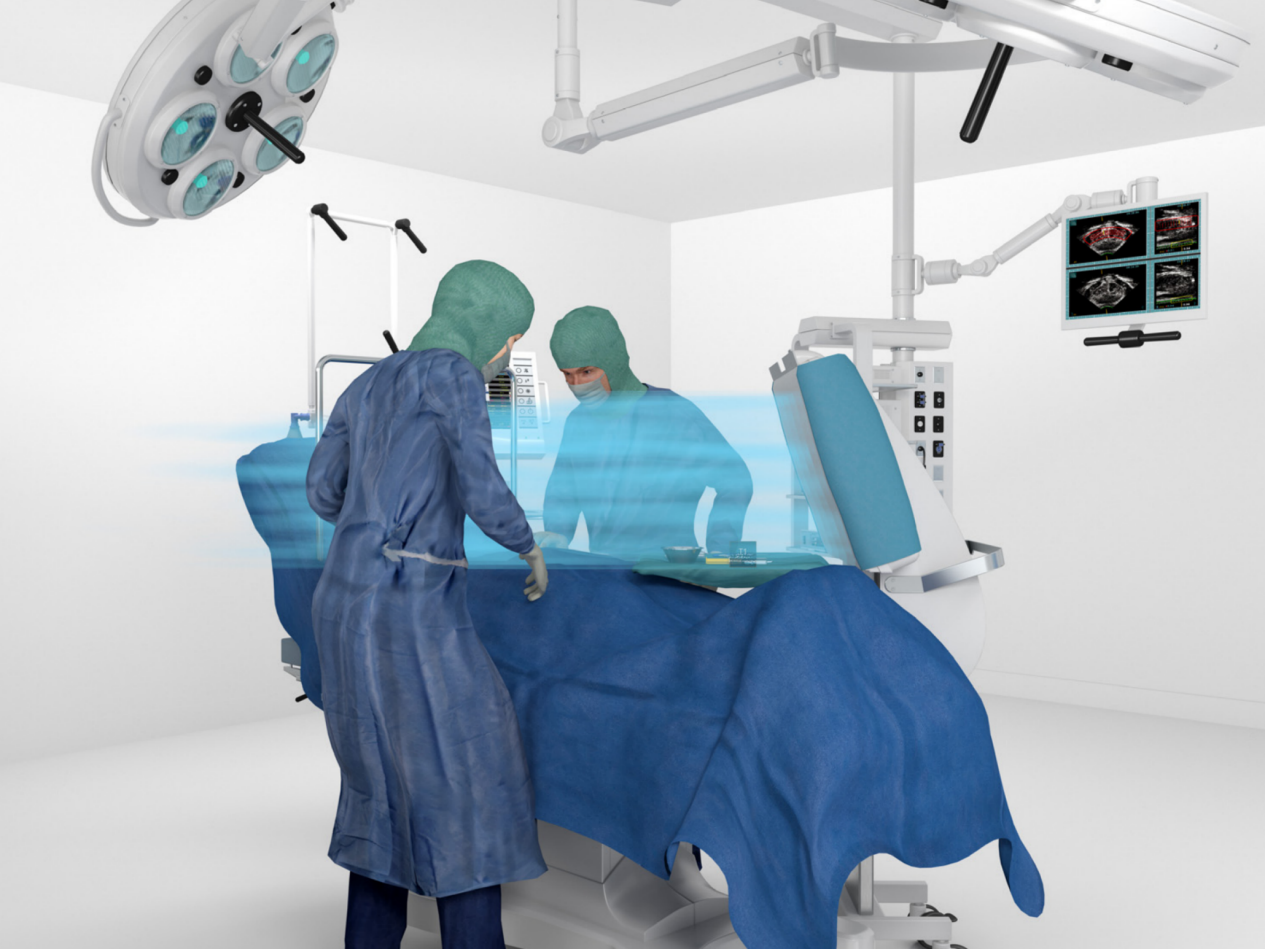
Breast surgery

**Figure 4:**
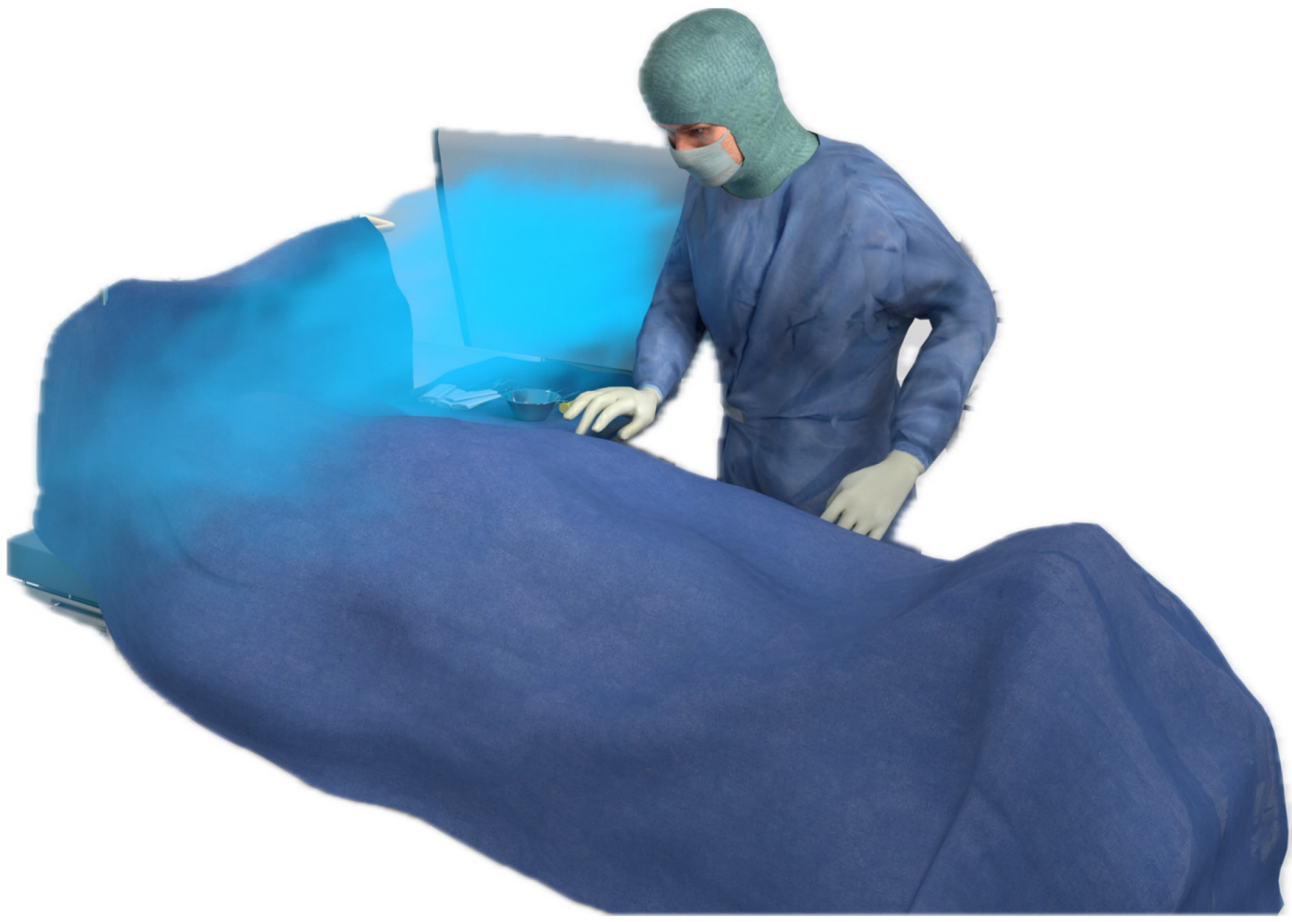
Heart surgery

**Figure 5:**
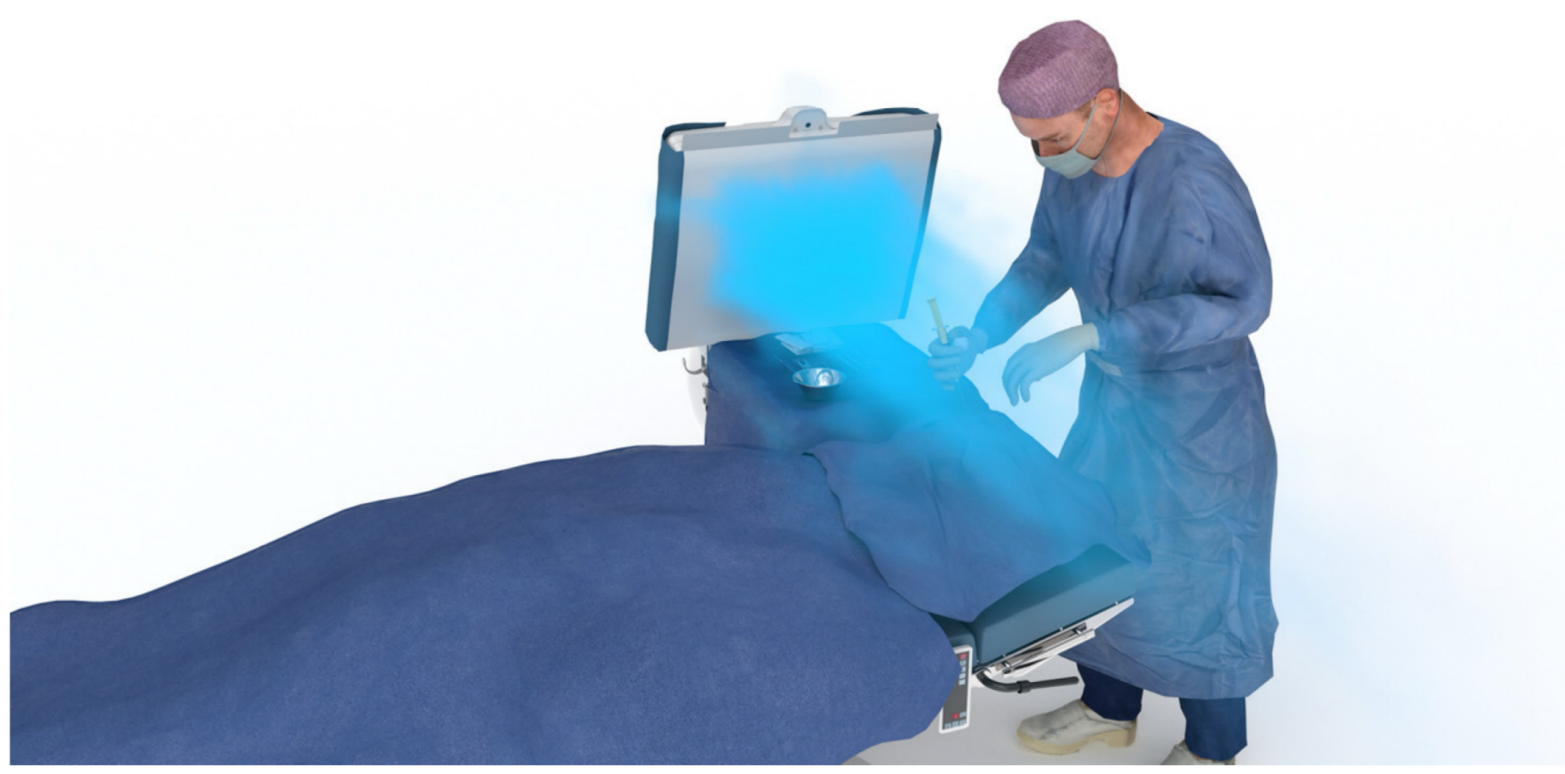
Surgery on the eye

Figure 2 (Fig. 2), Figure 3 (Fig. 3), Figure 4 (Fig. 4), and Figure 5 (Fig. 5) show schematically typical configurations for the use of the MSVU. 

### Performing the measurement in operation

While preparations were being made for the next operation, the MSVU was prepared by attaching the sterile shield while the flow was still switched off and then pressing the button that reads the barcode on the sterile shield. As soon as all indicators light up green, the unit is ready for use. At the start of the operation, the device was brought into position, i.e. the surgical field to be covered was set on the display, the air flow was switched on and 1 min was waited until the incision was made. The MSVU remained in operation during the entire operation and was only switched off at the end of the operation. The windows were closed continuously from the start of the preparations in the surgical unit until the end of the measurements.

Relative humidity and temperature were measured at the beginning and end of each operation. Agar plates were set up at four measuring points (M) during the operation to record the microbial load (Figure 1 (Fig. 1)):

- M1 and M2 were located on the instrument table near and far from the outlet of the MSVU, respectively. M1 was located in the right rear corner of the instrument table directly in front of the flow outlet. M2 was located on the opposite corner of the instrument table approx. 45 cm from the flow outlet (size of the instrument table 40 cm x 60 cm; Figure 1 (Fig. 1)).
- M3 was located next to the surgical field on the patient’s abdomen at a maximum distance of 25 cm from the surgical field (Figure 1 (Fig. 1)).
- M4 was located outside the sterilely ventilated area on the windowsill of the room approx. 135 cm from the operating field (Figure 1 (Fig. 1)).

Sterile-packed sedimentation plates were used for measuring points M1, M2 and M3 and placed on the measuring points by the operating theatre nurse. At measuring point M4, an aseptically packed sedimentation plate was placed by the doctoral student. The agar plates were opened at the beginning of the incision and left open until the end of the suture. 

**Figure 6:**
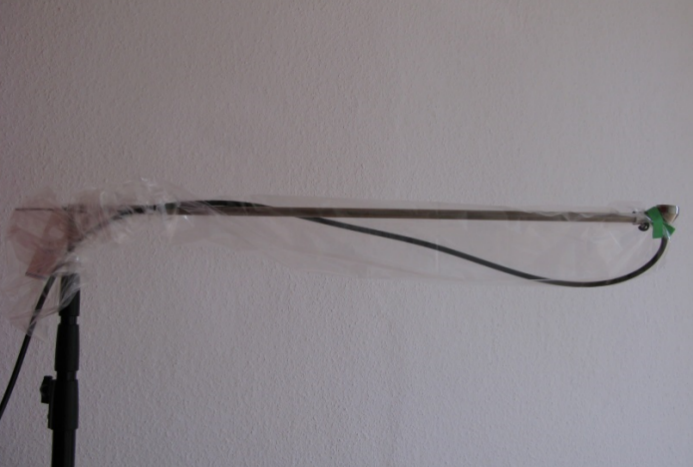
Structure of the particle measurement

The particle count (size fraction 0.5 µm–4.9 µm) was recorded continuously for a period of 1 min (corresponding to an air volume of 28.3 litres) over the period from the incision to the suture using the particle counter. For this purpose, the measuring probe of the particle counter was attached to a self-built frame and placed as close as possible to the surgical site (Figure 6 (Fig. 6)). The distance of the particle counter varied between 120 cm and 140 cm depending on the height of the surgeon, so that the measurement always took place approx. 30 cm above the patient just behind the surgical site. A tripod with a steel tube inserted into the centre served as a holding device. Another steel tube was attached to the steel tube via a sleeve at a 90° angle, at the end of which there was a screw clamp in which the measuring probe could be attached. The measuring probe and the steel tube were covered with a sterile camera cover and the camera cover was fixed to the opening of the particle filter with the supplied adhesive strip so that the opening was not covered. The funnel of the measuring device was sterilised in the steam steriliser of the operating unit at 134°C for 3 min holding time before each measurement. Before recording each measurement run, a zero adjustment was carried out using a particle-tight sterile filter to eliminate any particles still present in the system.

### Accompanying measurements

For each surgery, the number of people present, the number of door openings during the operation and the duration of the procedure (incision-suture time) were recorded. The latter was converted to 1 hour. The temperature and relative humidity were measured at the beginning and the end of the surgery in the centre of the operating theatre at a height of 120 cm above the floor.

### Data processing

The statistical programme IBM SPSS Statistics Version 28 (IBM Inc.) and Microsoft Excel 2010 were used for the statistical evaluation of the measured values. The significance level was set at p≤0.05. The test for normal distribution using the Kolmogorov-Smirnoff adjustment test only partially showed a normal distribution of the measured values, so that the Mann-Whitney U test for independent samples was carried out as a non-parametric test procedure. 

## Results

### Accompanying circumstances of the measurement situations

The number of people and the duration of the operation did not differ significantly between measurement situations A, B and C (Table 1 (Tab. 1)). In contrast, the number of door openings in measurement situation A was about twice as high as in measurement situations B and C. 

### Microbial indoor air pollution

The MSVU (measurement situation A) achieved a significant reduction in the number of sedimented CFU at measurement points M1, M2 and M3 compared to the values with the system switched off (measurement situation C). Only at measuring point M4 did the values not differ between the system being switched on and off. Although the number of door openings in measuring situation A was almost twice as high as in measuring situation B, significantly lower values were achieved in measuring situation A at measuring point M1 than in measuring situation B (Table 2 (Tab. 2)). The microbial load was reduced by 88.4% at measuring points M1, M2 by 91.5% and M3 by 65.2%. Even with an increased distance of 40 cm between the LAF and the instrument table (measurement situation B), the difference at measuring points M1, M2 and M3 was still significant compared to the switched-off system, albeit tending to be significantly lower at M1 (Table 2 (Tab. 2)).

Similarly, the number of sedimentation plates without bacteria detection differed. When the MSVU was switched off (measurement situation C), a higher number of plates with growth was detected at measurement points M1, M2 and M3 than when the system was operating properly (measurement situation A) (Table 3 (Tab. 3)). However, the difference was not significant due to the small sample size. 

There were only slight differences in pathogen detection between the 3 measurement situations (Table 4 (Tab. 4)). Tendentially, more coagulase-negative staphylococci (CoNS) were detectable when the MSVU was not switched on. Gram-negative bacteria were not detectable in any case. 

### Particle count in the room air

**Figure 7:**
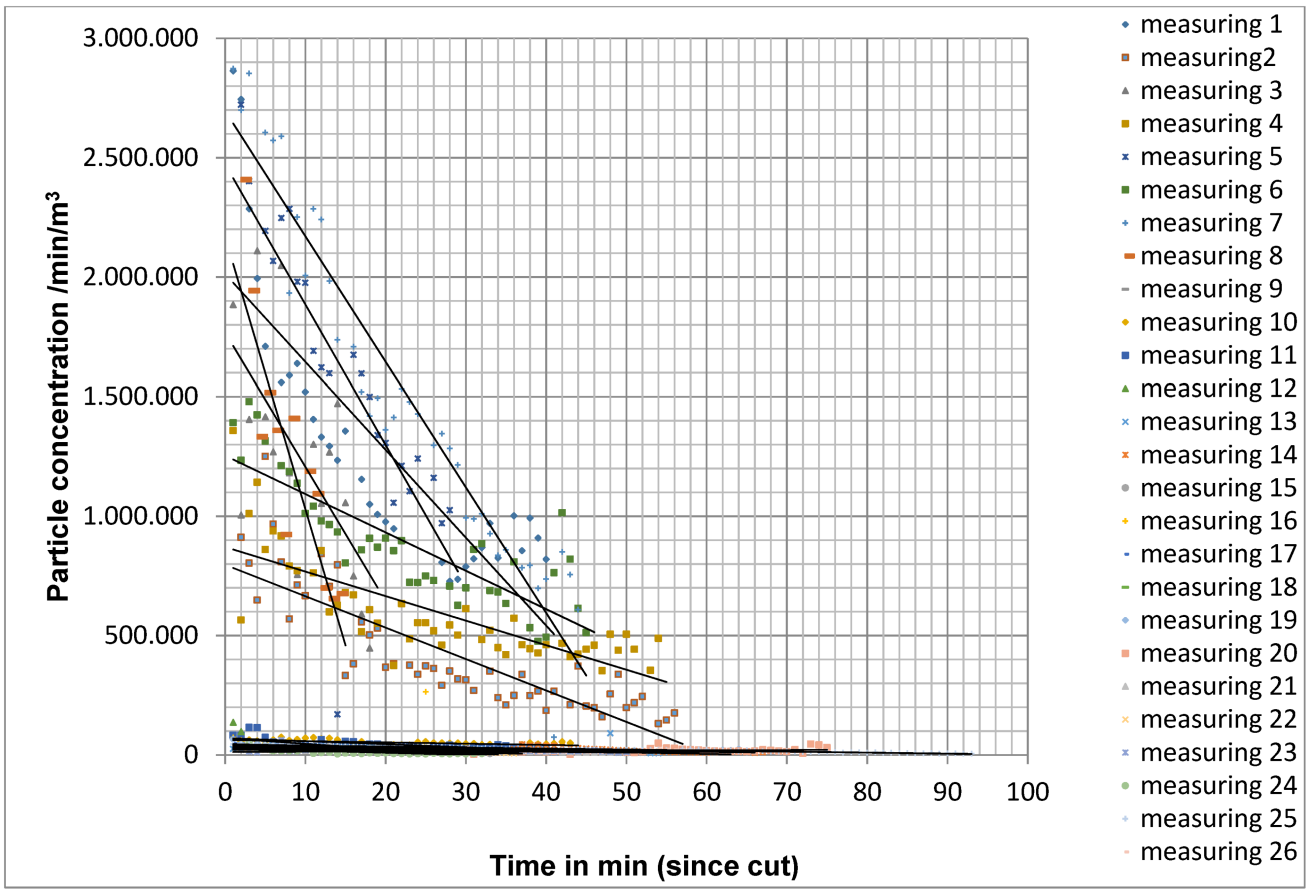
Decrease in particle concentration in measurement situation A for 26 operations

By continuously measuring the particle concentration at the measuring point, the particle concentration determined per minute was recorded over the entire course of the operation directly behind the operation area. In measurement situation A, the initial level of particle concentration at the beginning of the measurements was 1,665,339 particles/m^3^/min (mean value for 26 surgeries) and decreased over the course of the surgery to an average of 371,528 particles/m^3^/min (Figure 7 (Fig. 7)). The quotient of the particle count during the last measurement and the first measurement was on average 0.34±0.26, which corresponds to an average reduction in the particle count of 66%. 

**Figure 8:**
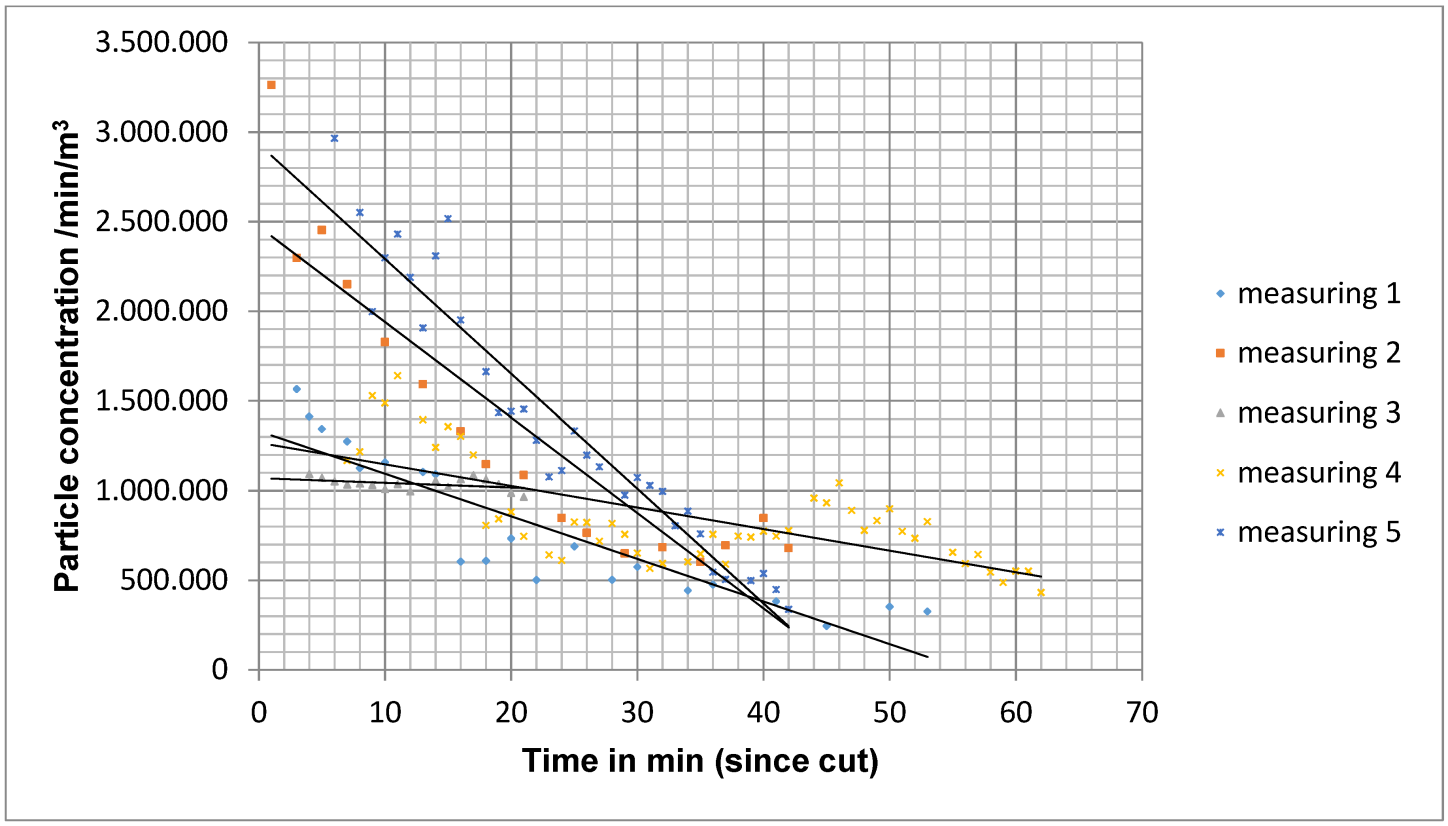
Decrease in particle concentration in measurement situation B with 5 operations

In measurement situation B, the initial level of particle concentration at the beginning of the measurements was 1,817,894 particles/m^3^/min (mean value for 5 operations) and decreased over the course of the operation to 548,051 particles/m^3^/min on average for all measurements. Compared to measurement situation A, the decrease in the particle count was significantly slower (Figure 8 (Fig. 8)). The quotient of the particle count during the last measurement and the first measurement was on average 0.36±0.28, which corresponds to an average reduction in the particle count of 64%.

**Figure 9:**
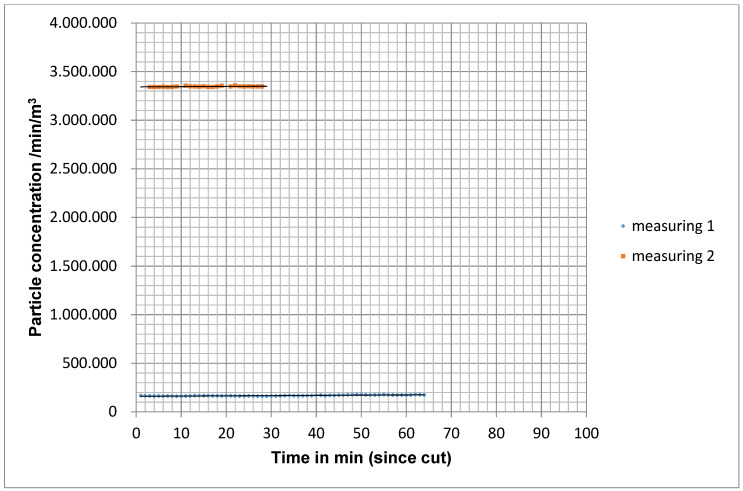
Course of the particle concentration with ventilation not switched on for 2 operations

When the MSVU was not switched on (measurement situation C), the particle count remained almost constant with a slight increase at the end of the operation (Figure 9 (Fig. 9)).

To statistically analyse the particle concentration over time, a bivariate linear regression line was determined from the particle concentrations measured from the cut to the suture. The slope of the straight line correlated with the drop in particle concentration. The frequency distribution of the slope did not show a normal distribution with the Kolmogorov-Smirnoff fit test. Therefore, the Mann-Whitney U test for independent samples was used as a non-parametric test method. In measurement situation A, the larger negative slope was detectable on average. The difference to measurement situation B was significant (p=0.003) with the exception of measurement point M4 outside the operating theatre field. Even between measurement situation B and C, the difference at measurement points M1, M2 and M3 was significant (p=0.048).

In situation A, a median reduction of the initial concentration by 50% was achieved after 15 min for particles of size 0.5 µm–4.9 µm. 

## Discussion

The studies on the role of RVS are based on the hypothesis that room air is a relevant reservoir of pathogens and represents a source for SSI. However, it should be noted that the main source of SSI are the patient's resident microflora which, depending on the location of the procedure, is released direct or indirect by translocation into the surgical wound i.e. from the nasal vestibule, the conjunctiva of the eye, the intestinal tract or from the skin adjacent to the surgical wound. The reason is that the resident flora cannot be completely eliminated by antiseptics. Thus the resident flora in the hair follicles [25], sebaceous and sweat glands are not reached by the preoperative skin antisepsis and only a part of the released resident flora, if the alcohol-based skin antiseptic contains a remanent additive, is subsequently inactivated, as alcohols themselves have no remanent effect [26]. In alloplastic joint implantations, vascular transplants and dermatosurgical procedures, the role of the skin flora as a source of SSI is obvious from the etiology, because representatives of the deep skin flora such as *Staphylococcus aureus*, CoNS, *Streptococcus* spp. and *Acinetobacter* spp. dominate as pathogens [27], [28], [29]. The release of resident skin flora into the surgical wound can also be recognised by the fact that at the beginning of the surgical procedure, sterile surgical gloves were contaminated with resident skin flora in 54% [30] at the time of insertion of the hip endoprosthesis and even in 91% at the time of insertion of vascular grafts [31]. In contrast, it is unclear how high is vthe proportion of bacteria released by the surgical team that can enter the surgical wound via airway. Hambraeus et al. [32] calculated from the amount of pathogens on surfaces and their number after redispersion into the room air that less than 15% of the bacteria detectable in the air in surgical units originated from the floor and categorised the risk of microorganisms stirred up from surfaces or floors as a source of SSI as low. However, as RVS are often required for large surgical units with continuous supply of fresh air with unknown microbial input, the ventilation requires sterile filtered supply air. Therefore RVS ventilated surgical units of RC Ib are considered the standard for surgieries with medium and high SSI risk [1]. However, there is a lack of evidence of infection prevention for RC Ib because it is not ethically justifiable to compare the SSI rate when performing the same procedures in RC Ib or RC II. A valid justification for RC Ib is that the high cost of reprocessing the sterile instruments would be reduced to absurdity if the instrument table in RC II were exposed to unprotected microbial contamination from the room air. In addition, an uncontrolled quantity of airborne microorganisms could enter the open surgical wound. Therefore, for operations with a medium and high SSI risk and in the case of serious consequences in the event of an SSI, RC Ib in the area of the operating field and the instrument table is considered standard [1]. If this can be achieved with an MSVU, both the costs and sustainability, but above all the horizontal overflow, speak in favour of using this instead of RVS.

The significant reduction in the microbial and particulate load in the room air in the aseptic working area is due to the MSVU, as the number of people in the operating unit and the duration of the operation did not differ between the 3 measurement situations and was even significantly higher when the system was switched on (Table 1 (Tab. 1)), the number of door openings was lowest in measurement situation C (Table 1 (Tab. 1)). Nevertheless, the number of CFU was significantly higher than when the ventilation system was switched on with a much higher number of door openings. The MSVU reduced the microbial load by 81.7% on average at measuring points M1, M2 and M3. With an RVS with TMF, the increased bacterial indoor air load caused by door openings was only reduced by 36.6% during ongoing operations [7]. Even if the spatial conditions and the range of operations differ, the result speaks for the efficacy of the MSVU.

The number of detectable CFU increased as the distance between the system and the operating theatre was increased. This illustrates that the correct positioning of the MSVU is important for its effectiveness.

The findings at measuring point M4 outside the overflow area make it clear that, as expected, the air flowing over the operating field only has an effect in the area of the instrument table and the operating field.

The results of air filtration and sedimentation measurements by Pasquarella et al. [33] are used to assess the results. The authors determined a different bacterial contamination of the room air depending on the risk area and recommended 5 CFU/sedimentation plate/hour as the maximum acceptable contamination for areas with very high requirements, e.g. in protective isolation, bone surgery operations and for the production of sterile drugs in the pharmaceutical industry, and 25 CFU/sedimentation plate/hour for areas with high requirements, e.g. in conventional operating units. For areas with high requirements, e.g. in conventional surgical units, 25 CFU/sedimentation plate/hour, for areas with medium requirements, e.g. kitchens and wards, 50 CFU/sedimentation plate/hour and for areas with low requirements 75 CFU/sedimentation plate/hour. Even if these values are not epidemiologically justified, they provide a realistic orientation based on the large number of values collected. With the MSVU, when optimally aligned to the operating field (measuring situation A) on the instrument table, the range for very high requirements was clearly undercut and the operating field was reached close (at M3). 

In measurement situation B, the mean values for M1 and M2 were also below 5 CFU/sedimentation plate/hour, but the range of dispersion exceeded the mean value of 5, while for M3 the mean value slightly exceeded the value of 5 CFU/sedimentation plate/hour. The range of 25 CFU/sedimentation plate/hour, as recommended for operations with a medium risk of developing SSI [22], was clearly undercut even at measuring point M4, even after switching off the RVS.

As expected, the microorganisms detected were Gram-positive representatives of the resident skin flora, which are released by the operating theatre team, and apathogenic spore-forming bacteria, which are ubiquitous in dust as environmental flora. Gram-negative bacteria with predominant origin from the intestine were not detectable in any case. 

The following comparison is instructive. For organizational reasons, a hospital wanted to swap an RC Ia operating theatre for an RC Ib operating theatre. By determining the sedimentation of microorganisms and particle measurement during regular surgery, the aim was to clarify whether the RC Ib operating unit achieves comparable results to the RC Ia operating unit. Both parameters were determined in 48 surgeries over the course of 7 weeks [34]. As both RVSs were operated in accordance with the standards resulting of testing at rest, the results allow a comparison with the effectiveness of the tested MSVU. For comparison with the MSVU, the measurement results for microbial sedimentation near the operating field and in the area of the instrument table as well as for the particle count near the operating field and in the area of the instrument table are considered for only RC Ib only, because its relevance for the comparison with the MSVU. With roughly comparable localization of the sampling points and identical measurement procedure, 2.6±2.9 CFU/50 cm^2^/hour sedimented in the area of the operating field and 4.7±4.6 CFU/50 cm^2^/hour upstream of the operating table [34], i.e. the values do not differ significantly from the values achieved with the MSVU (Table 5 (Tab. 5)). 

It is not known whether there is a critical particle number above which there is an increased risk of developing an SSI in RC Ib. Standards in this regard were drawn up by technicians without reference to the risk of infection based on infection epidemiology. In the surgical unit with RVS of RC Ib, a mean value of 117.669 particles was measured above the surgical field during gynecological operations (mean operation duration 50 min) [34] (Table 5 (Tab. 5)). Tendentially, the number of particles when using the MSVU is higher than In the surgical unit with RVS of RC Ib (Table 5 (Tab. 5)). But the values are not comparable with the MSVU situation because the RVS was running in continuous operation in the surgical unit, while the MSVU was only switched on 1 min before the incision. Even if the particle count is higher than in the study in RC Ib (Table 5 (Tab. 5)), this is not relevant from infectiological point of view, because it has only been shown for particles >5 µm that the number of sedimented CFU close to the surgical field increases with a higher particle number [34] (Table 5 (Tab. 5)). The effectiveness can presumably be improved if the MSVU is switched on 5 minutes before cutting instead of 1 minute, for example. For switching off RVS in operating theaters, significantly longer lead times of ≥20 min were set for recommissioning, depending on the system. Other parameters such as the type of draping, the sequence of preoperative steps, the positioning of the instruments and the type of protective clothing may have further, but probably not significant, influences. The separation of the operating field from the anesthesia area could also have an effect if the operating area is close. It can also be investigated whether mobile technology improves the working conditions for the surgical team.

The results obtained with the MSVU agree with previous studies. During orthopedic implant insertions (n=48), separate ventilation of the instrument table significantly (p<0.001) reduced the air contamination above the instruments compared to the ambient air (in 10 surgeries with and without mobile unit, respectively, indoor air pollution 0.3±0.78 CFU/m^3^/hour and 13.4±13.25 CFU/m^3^/hour [35]. Comparable results were achieved in two RC Ia surgical units by additional ventilation of the instrument table [36], [37]. In RC Ib, RC IA was achieved by additional mobile ventilation above the instrument table [38]. In a neurosurgical operating unit with turbulent ventilation (15 air changes/hour), the air colony count was significantly (p<0.001) reduced (<0.5 m away from the operating field 15.0 [7.5–43.5] and 2.0 [0.0–4.0] CFU/m^3^/hour) by additionally installing the mobile ventilation unit both in the operating area and above the instrument table [39]. In an analogue study of alloplastic knee implantation, the airborne exposure in the surgical field was reduced from 23.5 to 3.5 CFU/m^3^/hour, i.e. below the recommended value for RVS with TAV. In contrast, the values in the area of the instrument table remained unaffected [40]. In a study similar to ours, the particle count in cataract surgery was reduced by 79% from 0.5 µ, 81.7% from 1 µ and 90.8% from 5 µ particles [41]. The reduction was thus even higher than in our study, presumably because the operation is associated with less particle exposure. Textile fibres in the eye (lint fibres) occurred significantly less frequently (6/50 eyes vs. 0/99 eyes, p=0.014) [40].

As the requirement in Italy for intravitreal injection (IVI) to be performed in a surgical unit, the consequence during the COVID-19 pandemic would have been a drastic reduction in the number of feasible operations. Because in countries such as USA and Canada IVI may be performed in the ophthalmologist’s practice, in an Italian study IVI was performed under the conditions of an ophthalmologist’s practice using MSVU. In 5,678 IVIs performed, the complication rate was low at 0.0465%; endophthalmitis developed once (0.0176%) [42]; this was within the observed endophthalmitis rate of 0.008 to 0.092% [43]. Surgical costs were reduced by 26% per IVI [42]. Using a similar approach in the UK, no SSI occurred in 1,269 cataract surgeries after review after 2 weeks. This emphasizes the safety of MSVU, as a questionnaire survey in the UK in 1999/2000 showed an incidence of 0.14% for endophthalmitis [44]. 

## Limitations

Due to the determination of the CFU, the measurement of particle sizes >5 µm was omitted, especially as these are present in smaller numbers than smaller particles and are eliminated more effectively due to their size. As the MSVU was switched on 1 min before the start of the operation, but the particle reduction only reaches a median of 50% after 15 min, the 1 min represents a worst case with regard to particle reduction. It should be checked what influence the start-up e.g. 5 min instead of 1 min before cutting has on particle elimination.

A second limitation is that the elimination performance was only determined with 2 measurements compared to the system that was not switched on. In view of the low scattering of the measured values between the two measurements, however, it can be assumed that the measured values reflect real conditions.

Although the MSVU was only tested for two types of surgery, it can be assumed that its effectiveness is guaranteed regardless of the type of surgery if it is positioned correctly.

## Conclusion

The conclusion is that the mobile ventilation unit, when installed in an RC II operating theatre in the area of the operating field and the instrument table, achieves values that correspond with an RVS which generate RC Ib in terms of both microbiology and particle count. This means that operations with a high and medium SSI risk can be performed under these conditions. This conclusion can also be derived from the comparison with the results of a study in a surgical unit with RVS of Rk Ib [34]. 

## Further considerations

The great advantage of mobile sterile ventilation units is that the creation of aseptic working areas is decoupled from building structures by locally targeted displacement of room air contaminated with microorganisms and particles, i.e. mobile sterile ventilation units bring the sterile filtered air flow locally to the required areas instead of completely ventilating the unneeded environment in the entire room with TMF. 

The mobile technology enables use at any time in different locations, e.g. during

- operations, catheter interventions and port implantations,
- the treatment of burns and chemical lesions, if necessary with
- reverse suction,
- in intensive care units, e.g. for emergency interventions, emergency caesarean section and other unplanned interventions.

As only electricity and a small amount of disposable material are required for operation, mobile surgical units can be used in developing countries, in war situations and under disaster conditions. This means that injured people can be treated on site instead of being transported over long distances. The manageable dimensions, low weight and simple handling allow storage, transportation, provision and readiness for use in just a few minutes. Even non-professionals can be instructed graphically or by video in a matter of minutes.

As MSVU are significantly less cost-intensive to install, maintain and more sustainable in operation, they can be taken into account in future when planning new buildings or reconstruction projects in both outpatient and inpatient surgical centres in hospital hygiene risk assessment and planning with regard to the protection of the operating field and the instrument table against airborne infectious agents. Even in inpatient surgical centres with RVS, in which operations are performed that require highly specialised equipment or equipment used by several surgical disciplines and for which interdisciplinary cooperation may be necessary or in which an unplanned extension may occur after an initially limited procedure, e.g. after implantation of a heart valve, the additional use of an MSVU can be useful in order to provide a targeted horizontal flow of sterile filtered air over the operating field with the associated advantages. If very large wound areas need to be covered, it may be advisable to cover the instrument table and the patient with a separate unit. Complex and very large OR settings can be covered by using several mobile units. In any case, the importance of the correct positioning of the mStBE to protect the operating field and instrument table must be emphasized.

Particularly when using currently used robotic surgical assistance systems (e.g. Da Vinci, Hugo, Versius and Asensus system), large parts of the surgical field are covered by the robotic systems. Here, the directional ceiling-to-floor ventilation cannot safely and continuously flow over the operating field, and undirected turbulence is caused by the flow towards the robotic system. In this case, horizontal overflow with lateral supply of sterile filtered room air to the operating field is more favourable in perspective. Irrespective of this, when using robotic systems, it should always be ensured that all surfaces that enter the air flow of the RVS are disinfected daily by wiping to prevent the introduction of airborne dust deposits, as the robotic systems are generally only provided with sterile coatings on the components directly related to the patient, but not on the load-bearing axial parts of the central column that house the operating arms. 

Hospitals are undergoing fundamental change and it is difficult to predict what intervention processes will look like in detail in a few years’ time. In any case, the proportion of minimally invasive operations will continue to increase, even with additional adjuvant therapy combinations. If expensive and specialized technology can be dispensed with, shell concepts and rooms can not only be built much more cheaply (estimated at up to 40%), they can also be repurposed much more easily. It is necessary to free oneself from traditional structures, room concepts and facilities. There are alternatives to ceiling lights, for example; a surgeon can use glasses to link data with the real view. Will there still be a need for many monitors and operating room lights that illuminate the operating field rather than the internal site? The combination of innovative technology with stringent processes will not only further reduce SSI rates but also improve the quality of work. Such a novel approach potentially enables further follow-up steps that would need to be evaluated, such as

- relieving the strain on operators through modified protective clothing and
- optimization of coverage and the process sequence.

Since the number of outpatient surgical centres corresponds to the number of patients who can be cared for at home after surgery and the acquisition of nosocomial pathogens in the home environment is unlikely, it also makes sense from this point of view to increase the number of outpatient surgical centres. This is promoted by the possibility of using mobile sterile ventilation systems instead of RVSs.

In any case, it must be ensured that SSI are recorded, as the conclusions of the study are based on the recording of microbiological parameters and particle counts, but not on a comparison of SSI rates.

If air conditioning and fresh air supply are required in addition to aseptic conditions, this can be achieved with an additional installation which is less complex than a RVS. For example, the Operio Clinic system can supply up to 1,080 m^3^ of filtered, germ-free outside air per hour into the operating unit. The positive pressure also prevents contaminated air from neighbouring rooms from entering [45]. If surgical smog is to be removed, local extraction devices must be used in the same way as for operating theatres ventilated with RVS in order to capture the smoke at source. 

Based on a risk assessment, Attachment 1 provides an estimate for selected operations for which RC II appears to be sufficient. 

## Notes

### Author contributions

The authors Harnoss and Kramer contributed equally.

### Authors’ ORCID

- Axel Kramer: 0000-0003-4193-2149
- Julian-Camill Harnoss: 0000-0002-5197-0248
- Colin M. Krüger: 0000-0003-4976-870X

### Ethical approval 

This study was conducted after approval by the ethics committee of the University of Greifswald (internal registration number (BB 082/18). 

### Funding 

Association of Statutory Health Insurance Physicians (Kassenärztliche Vereinigung, KV) Mecklenburg-Pomerania.

### Acknowledgments 

We would like to thank the Association of Statutory Health Insurance Physicians for the financial support for the procurement of culture media for microbiological cultivation. We would also like to thank PD Dr. Volker Worm, Medical Care Centre for Surgery, Orthopedic and Traumatology, Greifswald, Germany.

### Competing interests

The authors declare that they have no competing interests.

## Supplementary Material

Recommendation for the allocation of selected operations to room class (RC) II based on the risk assessment for future planning

## Figures and Tables

**Table 1:**
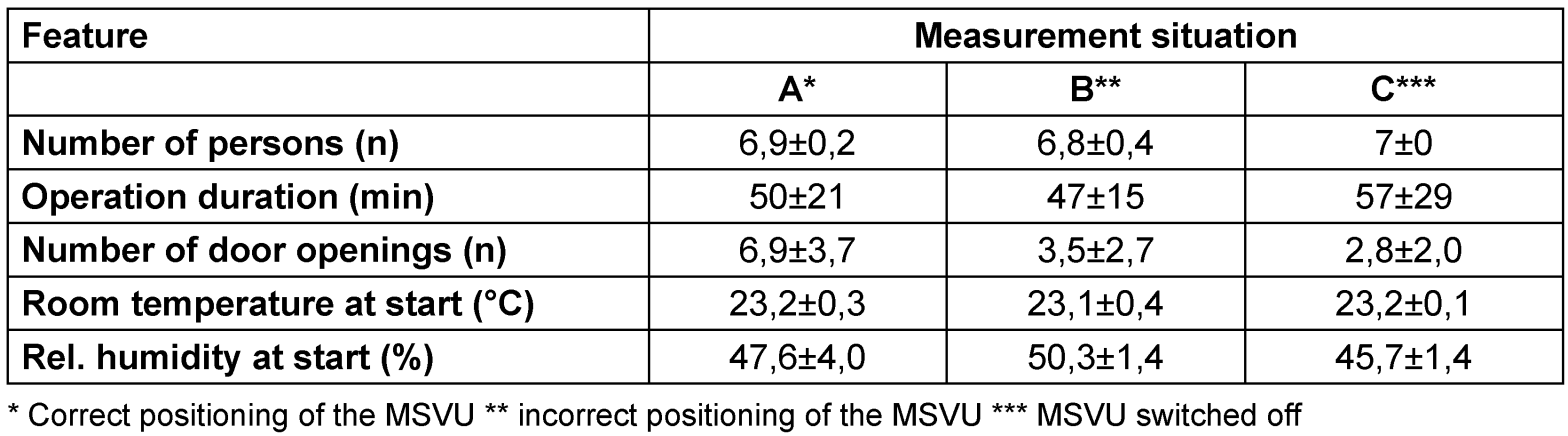
Frequency (mean value and dispersion) of the accompanying circumstances in the three measurement situations

**Table 2:**
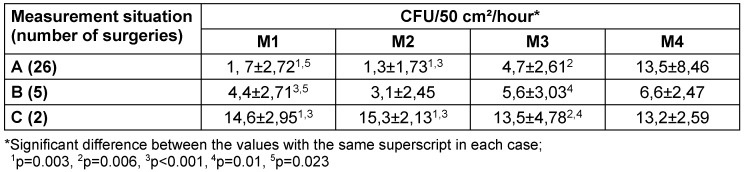
Results (mean value and scatter) of microbial sedimentation

**Table 3:**
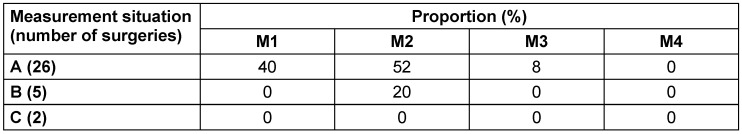
Proportion (%) of sedimentation plates without growth of CFU

**Table 4:**
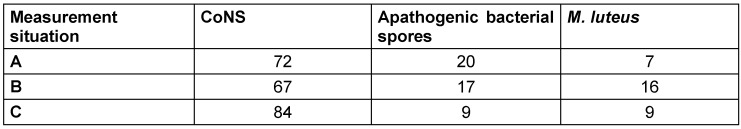
Proportion (%) of detected pathogens in the three measurement situations

**Table 5:**
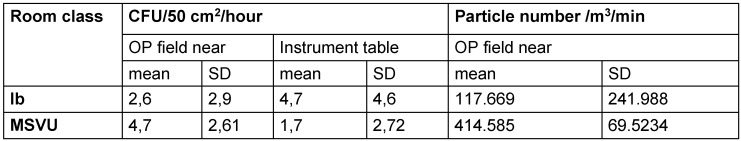
Mean values and standard deviation (SD) for the number of colony-forming units (CFU) per hour and for the mean particle number (0.5–4.9 µm) in the course of surgery

## References

[1] (2018). Prävention postoperativer Wundinfektionen: Empfehlung der Kommission für Krankenhaushygiene und Infektionsprävention (KRINKO) beim Robert Koch-Institut. Bundesgesundheitsblatt Gesundheitsforschung Gesundheitsschutz.

[2] Ahl T, Dalén N, Jörbeck H, Hoborn J (1995). Air contamination during hip and knee arthroplasties. Horizontal laminar flow randomized vs. conventional ventilation. Acta Orthop Scand.

[3] Hansen D, Krabs C, Benner D, Brauksiepe A, Popp W (2005). Laminar air flow provides high air quality in the operating field even during real operating conditions, but personal protection seems to be necessary in operations with tissue combustion. Int J Hyg Environ Health.

[4] Talon D, Schoenleber T, Bertrand X, Vichard P (2006). Performances en activité de différents types d'installation de traitement de l'air au bloc opératoire. Ann Chir.

[5] Diab-Elschahawi M, Berger J, Blacky A, Kimberger O, Oguz R, Kuelpmann R, Kramer A, Assadian O (2011). Impact of different-sized laminar air flow versus no laminar air flow on bacterial counts in the operating room during orthopedic surgery. Am J Infect Control.

[6] Hirsch T, Hubert H, Fischer S, Lahmer A, Lehnhardt M, Steinau HU, Steinstraesser L, Seipp HM (2012). Bacterial burden in the operating room: impact of airflow systems. Am J Infect Control.

[7] Smith EB, Raphael IJ, Maltenfort MG, Honsawek S, Dolan K, Younkins EA (2013). The effect of laminar air flow and door openings on operating room contamination. J Arthroplasty.

[8] Lidwell OM, Elson RA, Lowbury EJ, Whyte W, Blowers R, Stanley SJ, Lowe D (1987). Ultraclean air and antibiotics for prevention of postoperative infection. A multicenter study of 8,052 joint replacement operations. Acta Orthop Scand.

[9] Nelson JP, Murray WR (1977). The operating room environ- ment and its influence on deep wound infection.

[10] Lidwell OM, Lowbury EJ, Whyte W, Blowers R, Stanley SJ, Lowe D (1982). Effect of ultraclean air in operating rooms on deep sepsis in the joint after total hip or knee replacement: a randomised study. Br Med J (Clin Res Ed).

[11] Kakwani RG, Yohannan D, Wahab KH (2007). The effect of laminar air-flow on the results of Austin-Moore hemiarthroplasty. Injury.

[12] Bosanquet DC, Jones CN, Gill N, Jarvis P, Lewis MH (2013). Laminar flow reduces cases of surgical site infections in vascular patients. Ann R Coll Surg Engl.

[13] Ritter MA, Stringer EA (1980). Laminar air-flow versus conventional air operating systems: a seven-year patient follow-up. Clin Orthop Relat Res.

[14] van Griethuysen AJ, Spies-van Rooijen NH, Hoogenboom-Verdegaal AM (1996). Surveillance of wound infections and a new theatre: unexpected lack of improvement. J Hosp Infect.

[15] Miner AL, Losina E, Katz JN, Fossel AH, Platt R (2007). Deep infection after total knee replacement: impact of laminar airflow systems and body exhaust suits in the modern operating room. Infect Control Hosp Epidemiol.

[16] Breier AC, Brandt C, Sohr D, Geffers C, Gastmeier P (2011). Laminar airflow ceiling size: no impact on infection rates following hip and knee prosthesis. Infect Control Hosp Epidemiol.

[17] Hooper GJ, Rothwell AG, Frampton C, Wyatt MC (2011). Does the use of laminar flow and space suits reduce early deep infection after total hip and knee replacement?: the ten-year results of the New Zealand Joint Registry. J Bone Joint Surg Br.

[18] Zheng H, Barnett AG, Merollini K, Sutton A, Cooper N, Berendt T, Wilson J, Graves N (2014). Control strategies to prevent total hip replacement-related infections: a systematic review and mixed treatment comparison. BMJ Open.

[19] Bischoff P, Kubilay NZ, Allegranzi B, Egger M, Gastmeier P (2017). Effect of laminar airflow ventilation on surgical site infections: a systematic review and meta-analysis. Lancet Infect Dis.

[20] Houltz E, Erichsen-Andersson A, Björkander E, Grant P, Gustén J, Malchau H, Jivegard L, Liljegren A, Moonen J, Petzold M, Svanberg T, Svensson M, Sjövall H (2020). Effectiv eness of laminar versus turbulent airflow in operating theatres, with regard to risk for postoperative surgical infetions.

[21] NICE Hypothermia: prevention and management in adults having surgery.

[22] Berríos-Torres SI, Umscheid CA, Bratzler DW, Leas B, Stone EC, Kelz RR, Reinke CE, Morgan S, Solomkin JS, Mazuski JE, Dellinger EP, Itani KMF, Berbari EF, Segreti J, Parvizi J, Blanchard J, Allen G, Kluytmans JAJW, Donlan R, Schecter WP, Healthcare Infection Control Practices Advisory Committee (2017). Centers for Disease Control and Prevention Guideline for the Prevention of Surgical Site Infection, 2017. JAMA Surg.

[23] Nilsson KG, Lundholm R, Friberg S (2010). Assessment of horizontal laminar air flow instrument table for additional ultraclean space during surgery. J Hosp Infect.

[24] Sossai D, Dagnino G, Sanguineti F, Franchin F (2011). Mobile laminar air flow screen for additional operating room ventilation: reduction of intraoperative bacterial contamination during total knee arthroplasty. J Orthop Traumatol.

[25] Lademann O, Kramer A, Richter H, Patzelt A, Meinke MC, Czaika V, Weltmann KD, Hartmann B, Koch S (2011). Skin disinfection by plasma-tissue interaction: comparison of the effectivity of tissue-tolerable plasma and a standard antiseptic. Skin Pharmacol Physiol.

[26] Harnoss JC, Assadian O, Kramer A, Probst P, Müller-Lantzsch C, Scheerer L, Bruckner T, Diener MK, Büchler MW, Ulrich AB (2018). Comparison of chlorhexidine-isopropanol with isopropanol skin antisepsis for prevention of surgical-site infection after abdominal surgery. Br J Surg.

[27] Tande AJ, Patel R (2014). Prosthetic joint infection. Clin Microbiol Rev.

[28] Ajdler-Schaeffler E, Scherrer AU, Keller PM, Anagnostopoulos A, Hofmann M, Rancic Z, Zinkernagel AS, Bloemberg GV, Hasse BK, and the VASGRA Cohort (2018). Increased Pathogen Identification in Vascular Graft Infections by the Combined Use of Tissue Cultures and 16S rRNA Gene Polymerase Chain Reaction. Front Med (Lausanne).

[29] Saleh K (2019). Surgical Site Infections in Dermatologic Surgery-Clinical, diagnostic, and pathogenic aspects [Doctoral Thesis].

[30] Beldame J, Lagrave B, Lievain L, Lefebvre B, Frebourg N, Dujardin F (2012). Surgical glove bacterial contamination and perforation during total hip arthroplasty implantation: when gloves should be changed. Orthop Traumatol Surg Res.

[31] Meyer MJ, Hoene A, Weinrich M, Zwicker P, Kramer A (2022). Investigations on bioburden on surgical gloves during vascular surgery and consequences, presentation at the 27th North German Vascular Days of the North German Society for Vascular Medicine.

[32] Hambraeus A, Bengtsson S, Laurell G (1978). Bacterial contamination in a modern operating suite. 3. Importance of floor contamination as a source of airborne bacteria. J Hyg (Lond).

[33] Pasquarella C, Pitzurra O, Savino A (2000). The index of microbial air contamination. J Hosp Infect.

[34] Schuler H (2018). Microbial and particulate contamination of room air under low-turbulence displacement flow and turbulent dilution flow during operating theatre operation [Dissertation].

[35] Seth Caous J, Svensson Malchau K, Petzold M, Fridell Y, Malchau H, Ahlstrom L, Grant P, Erichsen Andersson A (2022). Instrument tables equipped with local unidirectional airflow units reduce bacterial contamination during orthopedic implant surgery in an operating room with a displacement ventilation system. Infect Prev Pract.

[36] Quint U, Benen T (2016). Mögliche Instrumentenkontamination im Operationssaal während der Implantation von Hüft- und Kniegelenkendoprothesen. Z Orthop Unfall.

[37] Friberg B, Lindgren M, Karlsson C, Bergström A, Friberg S (2002). Mobile zoned/exponential LAF screen: a new concept in ultra-clean air technology for additional operating room ventilation. J Hosp Infect.

[38] Sadrizadeh S, Tammelin A, Nielsen PV, Holmberg S (2014). Does a mobile laminar airflow screen reduce bacterial contamination in the operating room? A numerical study using computational fluid dynamics technique. Patient Saf Surg.

[39] von Vogelsang AC, Förander P, Arvidsson M, Löwenhielm P (2018). Effect of mobile laminar airflow units on airborne bacterial contamination during neurosurgical procedures. J Hosp Infect.

[40] Sossai D, Dagnino G, Sanguineti F, Franchin F (2011). Mobile laminar air flow screen for additional operating room ventilation: reduction of intraoperative bacterial contamination during total knee arthroplasty. J Orthop Traumatol.

[41] Osher RH, Figueiredo GB, Schneider JG, Kratholm J (2021). Purifying air over the operating field with a new mobile laminar airflow device to reduce the possibility of airborne contamination. J Cataract Refract Surg.

[42] Scarpa G, Urban F, Scarpa M, Formentini S, Beccastrini A (2022). Intravitreal Injections during the COVID-19 Outbreak in Northern Italy: An Innovative Approach for a High Quality and Safe Treatment. Eur J Ophthalmol.

[43] Cox JT, Eliott D, Sobrin L (2021). Inflammatory Complications of Intravitreal Anti-VEGF Injections. J Clin Med.

[44] Patel RP, While B, Smith A, Deutsch J, Scotcher S, Morphis G, Williams GP, Madge SN (2023). Initial experiences of cataract & lens surgery in 1269 patients in outpatient clean rooms using a portable laminar air flow device. Eye (Lond).

[45] Normeditec Room air technology (RLT) system in the operating theatre and operating theatre Operio CLINIC.

