# Supplementary material for: Achieving room air quality of room class Ib in the aseptic area using a mobile sterile ventilation unit in a room class II surgical unit
Source: GMS Hyg Infect Control. 2024 Dec 16;19:Doc66. doi: 10.3205/dgkh000521 (PMC11730182; doi:10.3205/dgkh000521)
Supplement: Recommendation for the allocation of selected operations to room class (RC) II based on the risk assessment for future planning [file HIC-19-66-s-001.pdf]

Attachment 1: Supplement

Recommendation for the allocation of selected operations to room class (RC) II based on the risk assessment for future planning

Regardless of their size and technical equipment, operating theatres ultimately only differ in the type of ventilation. According to the recommendation of the Commission for Hospital Hygiene and Infection Prevention at the Robert Koch Institute in Berlin [1], surgeries with a low or minimal SSI risk can be performed in an operating theatre without a room ventilation system (RVS). This recommendation results from a risk assessment based on SSI rates and their impact assessment, but cannot be justified epidemiologically because there is no comparison of SSI rates if similar operations with low or minimal SSI were performed in operating theatres without or with RVS. An additional criterion for risk assessment is the typical aetiology for a type of surgery. If it is predominantly Gram-positive staphylococci, these originate mainly from the patient's resident skin flora and only a small proportion is released by the surgical team. RVS is only expected to have an effect on the latter. If Gram-negative intestinal flora dominate as pathogens, the room air is not relevant as source of contamination. A further criterion for the decision in favour of an RVS is the level of defense, because the SSI risk increases in the case of severe immune deficiency, so that RC Ib in the aseptic working area may also make sense for procedures with a medium or low risk of an SSI.

Provided that a mobile sterile ventilation unit (MSVU) achieves a comparable reduction in the contamination by horizontal overflow of the aseptic working areas as an RVS with vertical supply of sterile filtered air, the decision in favour of one or other form of ventilation can be made dependent on other factors such as the required air conditioning, the space required for the provision of sterile instruments and sterile implants and an assessment of the extent to which flow obstacles in the air stream lead to turbulence. And if the MSVU is not sufficient to cover the aseptic storage area and the patient at the same time, this can be achieved with 2 separate MSVUs if necessary. During the planning phase, it must be agreed with the manufacturer whether an MSVU or an RVS is required.

In principle, a decision must be made on the basis of a risk assessment as to whether ventilation with triple-filtered so-called sterile air is required. In order to stimulate the formation of opinions on this internationally, Table 1 lists operations for which sterile ventilation appears to be dispensable. The criteria for the risk assessment are the antisepsis achievable by preoperative antiseptics, the extent of the surgical wound, the susceptibility to infection of the exposed tissue, the SSI rate and the microbial aetiology in the event of an SSI.

Independent of this, the definition and implementation of evidence-based pre-, peri-, intra- and postoperative infection prevention measures, ideally as a controlled bundle based on procedural instructions, is crucial for the prevention of SSIs.

Table 1: Suggestions for surgeries that can be performed in space class II (modified according to [2])

Speciality	Surgery
**Orthopaedics, surgery, neurosurgery**	Microsurgical reconstructive surgery on the finger or toe artery; neurolysis for carpal tunnel syndrome including nerve displacement and re-embedding; denervation of the wrist, finger or toe joint; end-to-end suture of a nerve in case of fresh injury; secondary suture of peripheral nerve by epineural suture; interfascicular microsurgical nerve suture also with defect bridging by graft; opening of osseous tendon sheath panaritium; treatment of palm phlegmon; surgery of a ganglion on a wrist, ankle or finger joint; extirpation of a tumour of the soft tissues of the fingers or toes; tendon sheath stenosis surgery of flexor/extensor tendon; tendosynovitis surgery in the wrist; removal of membranous connection between fingers or toes; muscle and/or fascia suture; dissection and suturing of an extensor tendon/flexor tendon, dissection and cutting of a tendon or muscle; shortening, lengthening or plastic excision of a tendon or fascia; removal of adhesions around a tendon; transplantation of a tendon or muscle; creation of a tendon bed; Dupuytren's contracture with partial removal of the palmar aponeurosis; carpal/tarsal tunnel syndrome; finger, toe or ankle joint and/or syndesmosis ligament reconstruction; removal of foreign bodies from the jaw, finger, wrist, toe or ankle joint; removal of Baker's cyst; synovectomy of finger/toe joint; removal of mucoid cyst; opening of finger, toe, jaw, wrist or ankle joint, if necessary including drainage; wire fixation to fix a small joint (finger or toe joint) or to fix several small joints or at the base of the thumb, metacarpus or metatarsus; stiffening of a finger or toe joint, arthroplasty of a finger, toe, jaw, wrist or ankle joint; resection of finger/toe joint; amputation or disarticulation of a finger or finger ray in the metacarpus; stabilisation of a fracture using percutaneous wire fixation; osteosynthesis of a small tubular bone, the collarbone, the olecranon, the kneecap, the inner or outer ankle; pseudarthrosis of the scaphoid, carpus or arthrodesis between two carpal bones; osteotomy and/or complete or partial resection of a small bone; removal of osteosynthesis material from a small and large tubular bone; trial gouging from a bone; bone gouging or necrotomy; resection of a small bone and, if applicable, of a neighbouring part of a joint; exostosis chiselling; surgical dislocation of the finger, thumb, toe, hand, foot or jaw joint; primary suture or reinsertion of a ligament and/or suture of the joint capsule of a finger or toe joint; eyelid surgery using skin flap displacement; anal fissure, including sphincterotomy if necessary including sphincterotomy; excision of haemorrhoidal nodes; segmental after Milligan-Morgan extirpation; reconstruction of a deep vein or artery opened by injury to the limbs by vascular suture; pilonidal cyst or fistula
**Aesthetic plastic surgery**	Eyelid surgery using skin flap displacement; otoclisis/otopexy/correction of auricle shape; full-thickness skin transplantation to cover a small skin defect; transplantation of hair-bearing skin implants; excision of large, function-impairing scars including plastic coverage; laser skin resurfacing

**Dermatosurgery**	Plastic surgery phimosis; cutaneous punch and incision biopsies; excision of nevus flammeus; opening of an abscess or excision of a carbuncle; microscopically controlled surgery of malignant skin tumours; local flap plasty after cutaneous single-stage tumour resection (small); skin transplantation (small); liposuction; endovenous laser therapy; monopolar electrodesiccation of anal intraepithelial neoplasia and condylomata acuminata; nail surgery; electrochemotherapy
**Ophthalmology**	IVI*; conjunctival tumours
**ENT**	Minimally invasive surgery to reduce snoring and improve nasal breathing; endonasal-endoscopic and microscopic surgery of the paranasal sinuses; laryngectomy (microlaryngoscopy); tonsillectomy; removal of polyps; tympanostomy tube insertion in children; hearing implant insertion; extirpation of acoustic neuroma; removal of auditory canal exostoses; marsupialisation, stone extraction, cervical punching and incisional biopsies; septoplasty, conchoplasty, conchotomy; high-frequency treatment of the turbinates; infundibulotomy; endonasal biopsies; osteoplastic Caldwell-Luc operations; cartilaginous septorhinoplasty; nasal inlet surgery; nasal bone reduction; adenotomy; panendoscopy; laser surgical scar cutting; laser-assisted or radiofrequency-assisted uvulopalatopharyngoplasty including modification without TE in the naso-, oro- and hypopharynx, oral cavity and oesophagus, oral mucosoplasty; removal of Reinke's oedema
**Gynaecology**	Minor invasive procedure in the vaginal-vulvar area; reduction of the labia minora

*Although endophthalmitis can occur very rarely as a very serious complication after IVI, antisepsis with an aqueous PVP-iodine solution covering all areas of the eye cannot be assumed to provide additional safety due to the overflow of sterile air.

For the following types of surgery, the risk assessment indicates the need for so-called sterile ventilation, i.e. room class Ib in the aseptic working area: procedures with a high or medium risk of SSI, open heart surgery, opening of large joints, peritoneum and pleura, insertion of large alloplastic implants, major high-risk procedures in the area of the brain, spinal cord, bone or spine, transplant surgery, removal of cartilage or bone material for free transplantation, implantation of cartilage or bone, large-scale skin transplantation

References

[1] Prävention postoperativer Wundinfektionen: Empfehlung der Kommission für Krankenhaushygiene und Infektionsprävention (KRINKO) beim Robert Koch-Institut. Bundesgesundheitsblatt Gesundheitsforschung Gesundheitsschutz. 2018 Apr; 61(4):448-73. DOI: 10.1007/s00103-018-2706-2

[2] Kramer A, Assadian O, Zacharowski K, Bulitta C, Vakil R, Lippert H. Klinische und ambulante Operationszentren, Herzkatheterlabor und Hybrid-Operationseinheit. In: Kramer A, Assadian O, Exner M, Huebner NO, Simon A, Scheithauer S, editors. Krankenhaus- und Praxishygiene Hygienemanagement und Infektionsprävention in medizinischen und sozialen Einrichtungen. München: Urban & Fischer (Elsevier); 2022. pp. 668-82.
